# Mindfulness on the Palm of Your Hand: A Systematic Review of Mobile Mindfulness Apps and Their Effects on Well-Being, Compassion, and Aggression in Non-Clinical Adults

**DOI:** 10.3390/healthcare14111512

**Published:** 2026-05-29

**Authors:** Félix Alberto Véliz-Montoya, Sandra Nieto-González, Antonio Salinas-Layana, Juan Pablo Pizarro-Ruiz

**Affiliations:** 1 Faculty of Education Sciences, University of Burgos, Calle de Villadiego, 1, 09001 Burgos, Spain; faveliz@ubu.es (F.A.V.-M.); rjpizarrpo@ubu.es (J.P.P.-R.); 2 Faculty of Psychology, University of Chile, Av. Capitán Ignacio Carrera Pinto 1045, Ñuñoa, Santiago 6850331, Chile; antonio.salinas@uchile.cl

**Keywords:** mindfulness, mobile applications, well-being, compassion, aggression

## Abstract

**Highlights:**

**What are the main findings?**

- Mobile mindfulness apps appear to be associated with small to moderate improvements in multiple dimensions of well-being, such as life satisfaction and positive affect, among non-clinical adult populations.
- A key research gap was identified: while well-being is widely studied, there is still limited empirical evidence regarding the effects of these digital tools on compassion, and no evidence was found addressing aggression in non-clinical samples.

**What are the implications of the main findings?**

- These findings suggest that mobile mindfulness apps may represent accessible tools for supporting psychological well-being in the general population, although the certainty of the evidence remains limited.
- At the same time, future research should expand beyond well-being indicators to further examine compassion and aggression outcomes, which remain underexplored in the current literature.
- The high variability in attrition rates indicates that technological availability alone is insufficient, highlighting the importance of incorporating personalized adherence strategies and culturally and linguistically adapted interventions (e.g., for Spanish-speaking populations) to support sustained engagement.

**Abstract:**

**Background/Objectives:** Mindfulness has emerged as a widely studied approach for promoting psychological well-being, evolving from its contemplative origins into a secular, evidence-based intervention. In recent years, the proliferation of mobile applications has enabled the delivery of mindfulness-based interventions (MBIs) in accessible and scalable formats. This systematic review examined the efficacy of MBIs delivered via mobile applications in non-clinical adult populations, with a focus on well-being, compassion, and aggression. **Methods**: A comprehensive search was conducted across six databases (Web of Science, PubMed, MEDLINE, Scopus, SciELO, and Dialnet) for studies published from 2014 onward, following PRISMA 2020 guidelines. A total of 23 randomized controlled trials met the inclusion criteria. Additionally, a risk-of-bias assessment was performed using the Cochrane RoB-2 tool. **Results**: Results indicated small to moderate improvements in well-being outcomes, including positive affect, life satisfaction, and psychological well-being. Evidence regarding compassion was limited and mixed, while no studies addressing aggression met the inclusion criteria. Additionally, substantial variability in adherence rates and a high overall risk of bias were observed. **Conclusions**: These findings suggest that mindfulness applications may represent accessible tools for enhancing individual well-being; however, their effect on broader socio-emotional functioning remains unclear. Further research should prioritize more rigorous study designs, including active control conditions and behavioral outcome measures, to better establish their effectiveness and underlying mechanisms.

## 1. Introduction

### 1.1. Origin and Theory of Mindfulness

Mindfulness has undergone a significant transition from its contemplative roots to become a validated psychosocial intervention strategy [1]. Originating from the Buddhist tradition, this term derives from the Pali word “*sati*” which refers to awareness, attention, and memory [2,3].

Despite this long journey and the fact that contemporary interest in mindfulness predates a wide range of contemplative strategies [4], its formal introduction to the West is attributed to Jon Kabat-Zinn [5]. Kabat-Zinn is notable for creating the Mindfulness-Based Stress Reduction (MBSR) Programme (formerly known as the Stress Reduction and Relaxation Programme) initially proposed as a tool for improving the symptomatology and mental health of patients with chronic pain; it is considered today a point of reference in the development of the so-called Mindfulness-Based Interventions or BMI [4,6,7,8].

Under this new paradigm, Kabat-Zinn [9] defines mindfulness as awareness centred on the present moment, characterized by an absence of judgement and criticism towards one’s own experience. However, despite the impact of this strategy in clinical settings and research [10], a lack of consensus in its definition persists, a situation that facilitates the indiscriminate use of the term by considering it a synonym for meditation equating its results with those of other meditative approaches, such as Zen or Vipassana meditation [4,8].

In line with the above, while the contemporary conception in the literature mostly follows Kabat-Zinn’s postulates [9], various authors have criticized the absence of an ethical component in the Western perspective [3]. For Buddhist philosophy, ethics is a fundamental pillar in the exercise of mindfulness; therefore, its exclusion could limit the potential benefits of traditional practices [3].

Conversely, others have warned of the risks of incorporating ethics into the scientific study of mindfulness, arguing that, given the complexity of the concept, its inclusion could compromise the internal validity of studies and the robustness of their conclusions [11].

In addition to the previous debate, contemporary psychology has also emphasized the various attitudes linked to mindfulness, requiring clarification of these elements before they are included in the study and practice of mindfulness [3]. Among the seven attitudes initially identified (non-judging, patience, beginner’s mind, trust, non-striving, acceptance, and letting go) [12], acceptance and non-judgement stand out by consensus for playing a fundamental therapeutic role by allowing an experience detached from desire, moral value, and hedonism [3].

Nonetheless, despite what has been described, the current landscape of mindfulness research reflects a lack of independent theoretical foundation, regardless of the relevance of its contributions [4]. In this direction, it should be mentioned that the present work is based on a secular and largely shared understanding of mindfulness as an innate quality capable of being refined through training [10]. Furthermore, following Kabat-Zinn’s approach [9], mindfulness is conceived as the awareness that arises from deliberate attention in the present moment, from acceptance, and in the absence of judgement towards the lived experience.

### 1.2. Foundations of Well-Being, Compassion, and Aggression

The study of well-being is another important link in the development of this work. In this regard, there is a dual perspective when understanding and analysing this concept: hedonic and eudaimonic [13,14]. The hedonic strand is oriented towards subjective well-being and is linked to happiness based on the prevalence of positive affective experiences; in contrast, the eudaimonic approach focuses on the individual’s personal growth, separating subjective well-being from psychological well-being [13].

In this direction, it is necessary to clarify that the literature includes various perspectives on what is understood by psychological well-being; for example, it can be analysed through six dimensions of functioning: self-acceptance, positive relations, environmental mastery, purpose in life, and personal growth [15]; understood as the search for “meaningful happiness” [16]; and, from a more recent perspective, linked to the satisfaction of three needs—competence, autonomy, and relatedness [17].

However, despite the debate over which paradigm is most suitable for studying this phenomenon, contemporary evidence suggests a multidimensional vision in which both approaches are not considered mutually exclusive but complementary, allowing for a holistic understanding of well-being [17]. Ultimately, this integral vision aims to encompass the complexity of the construct and shift the focus towards the mechanisms that sustain and explain this variable [14].

For example, compassion plays a role in psychological well-being, with a positive relationship observed between both factors, considering it an essential and relevant element for cultivating well-being [18]. We must not forget that compassion has been a potential component among traditional contemplative practices, with interest in its understanding and analysis increasing in recent decades [1,19].

Similarly to well-being, compassion has been approached from different perspectives to identify the core processes and attributes that define it [19,20]. From an evolutionary and motivational perspective, six factors associated with this construct have been identified: sensitivity to perceive and respond to the needs of others; sympathy or concern for others; and empathy, understood as the faculty to put oneself in another’s place [21]. Added to these are the motivation to alleviate suffering; distress tolerance, which protects from identification with another’s pain; and non-judgement, which allows for a compassionate approach free of evaluations [21].

Some authors, deepen the emotional component, by understanding compassion as an affective state or trait that differs in form and purpose from emotions such as love, distress, and sadness [22]. Finally, there is some agreement in pointing out that compassion involves an awareness of another person’s suffering, accompanied by a motivation to dispel it [20].

Lastly, aggression is considered due to its high impact and social cost [23]. As a phenomenon inherent to the human condition that transcends eras and cultures, it is defined as an intentional behaviour oriented towards causing harm to others [24]. In this regard, the General Aggression Model (GAM) is proposed, which understands aggression through the activation of cognitive schemas stored in memory and aims to be a useful and far-reaching approach when incorporating advances in the study of the aggressive response [24].

Furthermore, the importance of including this factor, beyond social impact, lies in its relationship with the previously mentioned variables (well-being and compassion). In this regard, a continuum between aggression and compassion is proposed to explain socio-emotional functioning, grouping various cognitive-emotional and behavioural responses that fluctuate across two large quadrants or levels: supportive and oppositional [25].

In addition, other studies have highlighted a negative relationship between aggression and well-being, noting how aggressive responses impact individuals’ development and mental health, which in turn affects emotional regulation and psychological well-being [26]. There is also substantial evidence supporting the effects of mindfulness on subjective well-being by favouring contact with positive experiences and promoting greater gratitude and life satisfaction [27]. Complementarily, other authors have emphasized the potential of mindfulness to promote psychological well-being, social connection, and self-esteem [28]. Recent studies have supported the utility of MBIs as ideal strategies for well-being care [29,30].

### 1.3. The Apps’ Era

The integration of mobile technologies in the healthcare field constitutes a strategic response to the global challenge in the promotion and prevention of well-being, and these applications (apps) are considered a potentially efficient solution to mitigate the gap linked to healthcare access [31]. Currently, the market offers various apps for self-care, which are useful not only for symptom reduction but also for fostering greater autonomy and empowerment among users [32].

Compared to traditional interventions, these tools stand out for their low cost, availability, and accessibility [33]. However, although some have demonstrated efficacy in improving well-being and mental health [32], it is imperative to deepen the understanding of the evidence and technical feasibility of such resources [31]. Within this new ecosystem, meditation applications, including those for mindfulness, have proven dominant in the digital mental health market [34].

This novel training format has results supporting its comparative efficacy with face-to-face interventions [35,36], even reporting lower attrition rates and an increase in time dedicated to practice [37]. However, the evidence is not unanimous. When contrasting the effects in students of an isolated MBI versus its combination with an app, no significant differences were found between the two modalities [38]. Furthermore, a positive relationship was observed between app use and the stress reported by participants. Given these findings, the need to continue studying the role of mindfulness apps remains clear, analysing factors that influence both their efficacy and user adherence.

Nevertheless, some works highlight the benefits of mindfulness practice, either face-to-face or via an app, on compassion and the reduction in aggression [39,40,41,42]; however, to date, no article has been found that collects evidence regarding the use of mindfulness applications for these variables in non-clinical populations.

Accordingly, this systematic review aimed at synthesising the available empirical evidence on mindfulness-based interventions delivered through mobile applications in non-clinical populations. While previous research has primarily focused on the effects of mindfulness interventions on well-being, less attention has been given to the impact on socio-emotional processes related with well-being such as compassion and aggression. In this regard, the study adopts a targeted approach by focusing on mindfulness apps as a central intervention, thereby distinguishing their specific impact from that of broader, multi-component programs [excluding approaches primarily grounded in loving-kindness, Compassion-Focused Therapy (CFT), or Mindful Self-Compassion (MSC)].

### 1.4. Objectives

The specific objectives of this study are as follows: (1) Integrate evidence regarding the efficacy of MBIs (apps) on well-being in non-clinical populations. (2) Collect available evidence on the impact of MBIs (apps) on aggression and compassion responses towards others. (3) To explore participant adherence and engagement patterns in mobile mindfulness interventions.

## 2. Materials and Methods

### 2.1. Search and Eligibility Criteria

The search was conducted between November and December 2024 using highly relevant indexed databases, including Web of Science, PubMed, MEDLINE, Scopus, SciELO, and Dialnet, to cover the literature in both English and Spanish. Search strategies were designed using a combination of Boolean operators and descriptors associated with mindfulness (Mindful*), the use of mobile devices (“mobile” OR “app” OR *phone), and methodological design (“RCT” OR “randomized control trial” OR “randomised control trial” OR “randomized clinical trial” OR “randomised clinical trial” OR “randomized controlled trial” OR “randomised controlled trial”)

It should be noted that the search string was adapted to the specific syntactic requirements of each database, and, following previous works [43,44], a time window from 2014 onwards was established. This delimitation is underpinned by the Mani study [43], which reported that, as of June 2014, the study by Howells [45] (published online) was the only randomised controlled trial evaluating the efficacy of a mindfulness application (Headspace). Therefore, establishing this timeframe enables the capture of the period of exponential growth in the scientific literature concerning the study and development of mindfulness apps, which, in recent decades, have increasingly assumed a prominent role as digital health tools.

### 2.2. Data Selection and Extraction

Selection was performed independently by two experts, with a third reviewer mediating to resolve discrepancies. The process was structured in three stages: first, a pilot phase with 30 random articles to evaluate titles, abstracts, and keywords according to the inclusion criteria (Table 1). After reaching a Kappa index of 0.82, a second phase of screening was conducted following the previously described procedure. Subsequently, in a third stage, each expert exhaustively examined the full text of the pre-selected articles to ensure strict compliance with the criteria before proceeding to data extraction, yielding a Kappa of 0.79. Additionally, Zotero 7 was used to manage the included references.

Next, for the final extraction, a new pilot was executed based on three randomly selected articles, which helped systematise the process among the evaluators. In both the pilot and definitive extraction, a pre-prepared digital spreadsheet (Microsoft Excel) was used following the PICO guiding framework. This framework structured the data extraction by categorising the study population, the intervention (MBI app), the comparative group, and the outcomes or measurement variables, thereby enabling the standardisation of the collected information and ensuring the replicability of the analysis (Table 2). During these stages, a third evaluator again resolved any disagreements. It is worth mentioning that this protocol was designed following the PRISMA 2020 statement guidelines (Supplementary Materials) [46].

Although the search yielded a total of 1648 records, after the detection and removal of duplicates using the EndNote manager 21 (Clarivate), the corpus was reduced to 1276 articles, which were exported for screening to Rayyan platform (Qatar Computing Research Institute). Additionally, following a reviewer’s advice, a cross-reference search was performed to ensure maximum coverage of the available literature. After this new search, four articles were included, yielding a total of 1280 studies for analysis.

## 3. Results

Of the 1280 potential works, 1256 were excluded after applying the eligibility criteria. The main reasons for exclusion included studies that did not assess at least one of the target variables—well-being, compassion, or aggression (*n* = 176); studies conducted with clinical populations or participants under 18 years of age (*n* = 305); interventions in which mindfulness was implemented only as a complementary component of a broader *programme* (*n* = 277); and study protocols or designs lacking at least two randomized comparison groups (*n* = 299). In addition, secondary studies, including scoping reviews, systematic reviews, and meta-analyses, were excluded (*n* = 200). This selection process was intended to ensure a methodologically rigorous final sample.

**Figure 1:**
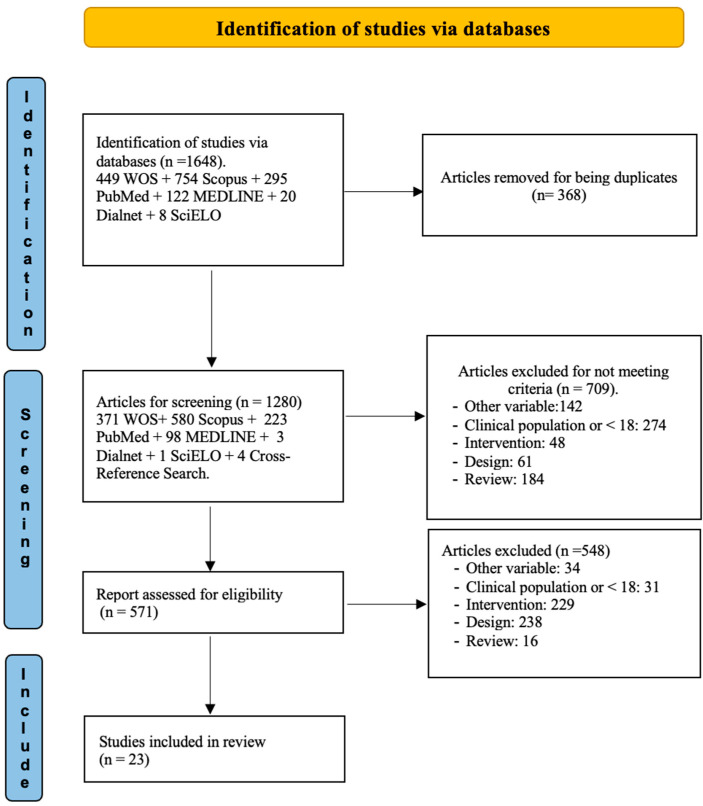
PRISMA flow diagram representing the selection process.

A total of 23 studies met the inclusion criteria and were included in the review. Most of the selected studies examined the effects of mindfulness-based interventions delivered through mobile applications on well-being outcomes (*n* = 21). Only one study focused exclusively on compassion-related responses, while one additional study assessed both well-being and compassion. No eligible studies examining aggression-related outcomes were identified (Figure 1); this absence of findings is primarily attributable to the study’s inclusion criteria, which strictly limited the scope to non-clinical populations. Whilst research in this field is emerging, it remains sparse. Although research on mindfulness apps and aggression-related outcomes is emerging, the available literature remains scarce. At the time of this review, only one study examining the effects of the Headspace application on aggression and impulsivity was identified, and it was conducted within a psychiatric population [42].

Overall, the findings suggest that mobile mindfulness interventions are generally associated with improvements in psychological well-being across different non-clinical populations and contexts. However, the evidence regarding compassion-related outcomes remains limited, and no empirical evidence was identified concerning aggression.

Most studies reported positive effects on dimensions such as life satisfaction, positive affect, flourishing, subjective vitality, and eudaimonic well-being following interventions delivered through applications such as Headspace, Insight Timer, InMind, and Living with Heart [49,51,54,67].

Several studies suggested that relatively brief interventions (e.g., 10–30 days) may be sufficient to produce improvements in affective well-being, particularly increases in positive affect [45,51]. Moreover, some evidence indicated that these benefits may persist over time, with sustained improvements reported up to three months post-intervention [58].

The effectiveness of mindfulness apps was also observed across diverse contexts, including occupational, academic, healthcare, and perinatal settings. In workplace populations, interventions were associated with improvements in psychological functioning, perceived well-being, and emotional regulation [47,63,65]. Similarly, positive outcomes were reported among university students and pregnant women, although findings in academic settings appeared more heterogeneous [48,66].

Despite these generally favorable findings, several studies reported null or modest effects. Some investigations failed to detect significant changes in subjective well-being, flourishing, or affective states compared to control conditions [55,59,62]. Additionally, some authors highlighted small effect sizes and limited robustness of findings [56].

Beyond well-being, only a limited number of studies explored prosocial variables such as compassion. Preliminary evidence suggested that mindfulness app use may promote compassionate responses, although the findings present certain discrepancies [41,50]. Table 2 presents the main characteristics and findings of the included studies.

### Risk of Bias

**Figure 2:**
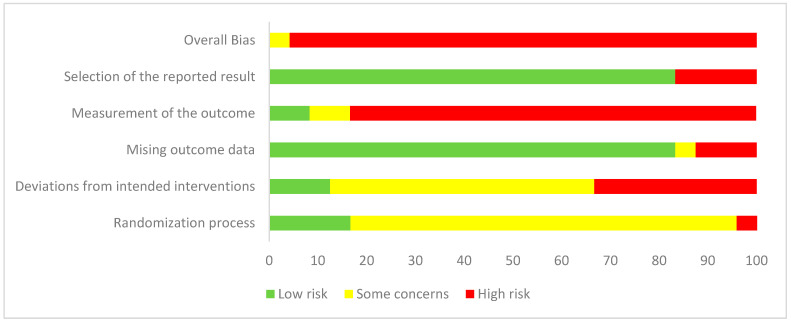
Cochrane risk of bias summary of the included studies.

The Cochrane RoB-2 tool [68] was utilised to estimate the risk of bias analysis (Figure 2). Regarding the randomisation process, 16.7% of the studies were considered at low risk, while 79.2% of the cases failed to report on the use of strategies intended to control for the potential effects of allocation. Despite the fact that many authors refer to the random allocation of participants to different study arms, the absence of methodological information could compromise the integrity of the findings.

Furthermore, the analysis of deviations from the intended interventions reveals that 33.3% of the articles exhibited a high risk of bias. This was largely ascribed, in most cases, to the use of waiting lists as control groups, wherein participant expectations may bias the results. Conversely, 12.5% of the papers mitigated this factor through the employment of active controls (such as sham meditation or cognitive tasks) blinding strategies, and the application of robust analyses to account for potential derived effects.

Even though attrition rates are a recurrent issue in this field, 83.3% of the papers reported the use of statistical techniques aimed at mitigating potential attrition effects, thereby estimating the impact of the intervention across the majority of participants. Nonetheless, 12.5% of the studies drew their conclusions exclusively from per-protocol analyses, which could overestimate the efficacy of MBIs (apps) by omitting those subjects who withdrew from the intervention before its completion. Regarding reporting integrity, 83.3% of the articles demonstrated a low risk of bias; however, a lack of concordance between the conducted analyses and the initial objectives persists in 16.7% of cases. Additionally, 83.3% of the papers presented potential limitations in their assessment, as they based their measurements on self-reports without blinding of the assessors.

In conclusion, the overall risk of bias among the included studies was high (83.3%). This indicates that, while mindfulness interventions (apps) show great potential, current scientific evidence requires addressing potential methodological limitations that compromise the quality of the available evidence.

## 4. Discussion

The present review synthesized the available evidence regarding the effects of app-based Mindfulness-Based Interventions (MBIs) on well-being, compassion, and aggression in non-clinical populations. Overall, the findings suggest that mindfulness interventions delivered through mobile applications are generally associated with improvements in several indicators of psychological well-being, including positive affect, life satisfaction, flourishing, and subjective vitality. Moreover, some studies such as that of Lindsay [57] have demonstrated that even a brief mindfulness practice (app) can be associated with a positive impact on participants’ well-being. These findings support the growing interest in mobile mindfulness applications as accessible and scalable tools for promoting mental well-being in everyday contexts.

Importantly, the well-being outcomes reported across studies can be differentiated into hedonic and eudaimonic dimensions. Hedonic well-being, typically operationalized through indicators such as positive affect and life satisfaction, was the most frequently assessed outcome and showed consistent but generally small to moderate improvements following app-based mindfulness interventions. In contrast, eudaimonic well-being, reflected in constructs such as flourishing and subjective vitality, was less frequently examined but also showed positive trends across several studies. This imbalance suggests that current research has placed greater emphasis on immediate affective outcomes, whereas more complex and longer-term dimensions of psychological functioning remain comparatively underexplored.

In this regard, one of the main contributions of the present review lies not only in confirming the potential association between app-based mindfulness practice and well-being, but also in identifying an important imbalance within the current literature. While previous studies and reviews have predominantly focused on well-being-related outcomes, considerably less attention has been paid to the possible effects of these interventions on socio-emotional variables such as compassion and aggression. In this regard, the present review extends the scope of the previous literature by specifically examining these less explored dimensions.

The results clearly reflect this asymmetry. Of the 23 studies included, 21 analyzed well-being outcomes, whereas only two examined compassion-related variables and none assessed aggression in non-clinical populations. This finding is particularly relevant considering that theoretical models of mindfulness frequently propose links between mindfulness practice, emotional regulation, prosociality, and reductions in reactive or aggressive behavior. Consequently, the available evidence appears to remain disproportionately centered on intrapersonal functioning, with limited investigation of interpersonal and socio-emotional processes.

One possible explanation for this imbalance may be methodological in nature. Well-being is commonly assessed through brief self-report instruments that are relatively easy to administer in digital contexts. In contrast, variables such as compassion and aggression often require more complex behavioral, observational, or ecologically valid assessment procedures, which are less frequently incorporated into app-based study designs. In this regard, the study by Lim [41] represents an important methodological contribution, as it evaluated compassionate responding through a naturalistic behavioral paradigm rather than relying exclusively on self-report measures. This approach remains uncommon within the current literature.

The evidence regarding compassion-related outcomes was also heterogeneous. While Lim [41] reported increases in compassionate behaviors following mindfulness app use, other studies found weaker or non-significant effects [50]. These inconsistencies may be explained by differences in intervention duration, adherence levels, intensity of practice, or the specific mindfulness components emphasized during training, such as attentional monitoring or acceptance-based processes. Additionally, compassion may constitute a more complex and temporally distal construct than immediate affective outcomes. Therefore, although brief interventions may be sufficient to generate short-term improvements in emotional regulation or positive affect, longer or more intensive practice may be necessary to produce stable changes in prosocial functioning.

At the same time, the absence of studies examining aggression-related outcomes in non-clinical populations represents a significant gap in the literature. Previous theoretical and empirical research has suggested that mindfulness may reduce aggressive responding by decreasing automatic emotional reactivity and strengthening self-regulation capacities. Nevertheless, the lack of studies conducted within digital and non-clinical contexts prevents conclusions regarding whether these mechanisms are effectively transferred to mobile mindfulness interventions.

Another important issue identified in this review concerns the methodological quality and potential risk of bias of the included studies. Several investigations presented limitations associated with small sample sizes, short intervention periods, reliance on self-report measures, and high attrition rates. Participant dropout varied substantially across studies, ranging from 4% to 70%, which may compromise both internal validity and the stability of the reported effects. Furthermore, many studies lacked active control groups, increasing the possibility that some observed improvements may be partially explained by expectancy effects, motivation biases, or non-specific intervention factors. Differences in adherence may also be influenced by specific study characteristics, such as intervention duration, target population, and type of application used, suggesting that these factors may play a moderating role in engagement outcomes.

The predominance of self-report methodologies constitutes an additional limitation within the field. Although validated questionnaires provide useful information regarding subjective experiences, they remain vulnerable to social desirability bias, shared method variance, and inaccuracies in self-perception. Consequently, the current evidence base may either overestimate or underestimate the real impact of mindfulness applications on everyday functioning. Future research would therefore benefit from incorporating multimethod assessment strategies, including behavioral paradigms, ecological momentary assessment, passive digital indicators, and longitudinal follow-up designs capable of capturing sustained behavioral change over time.

However, although the literature shows a clear interest in understanding these innovative strategies, a predominance of English-speaking applications is observed, alongside a deficit of studies aimed at validating these digital MBIs in Spanish-speaking populations. This limitation restricts the cultural generalizability of current findings and highlights the need for linguistically and culturally adapted mindfulness applications capable of responding to the needs of more diverse populations.

Among the limitations of this study, firstly a notable limitation of the evidence base is the predominance of studies using the Headspace application, which was employed in 15 of the 23 included studies. This concentration limits the generalizability of the findings to other mindfulness-based applications and should be considered when interpreting the results. Also, it should be acknowledged that the review did not include a meta-analysis, which precludes a quantitative synthesis of the results. Furthermore, the exclusion of grey literature may have introduced a degree of publication bias. Finally, a formal assessment of the certainty of the evidence was not conducted, which should be considered when interpreting the findings.

Consequently, it is recommended that these data be treated with caution. The lack of studies involving non-clinical populations that address the impact of MBIs on aggression limits the scope of this analysis. This underscores the need to examine the effects of these interventions beyond general well-being and symptom reduction, with particular attention to the underlying mechanisms that may explain their outcomes.

### Practical Implications

From an applied perspective, these findings suggest that mindfulness apps should be considered as complementary tools rather than stand-alone interventions within clinical and preventive settings, particularly when access to face-to-face care is limited. For developers, the results highlight the importance of improving adherence and engagement through user-centered design, culturally and linguistically adapted content, and features that support sustained practice over time. For healthcare systems and policymakers, cautious implementation is recommended, ensuring that the use of these applications is supported by independent evaluation, clear standards of evidence, and transparent communication regarding their level of empirical support.

## 5. Conclusions

Mindfulness apps appear to represent accessible and scalable low-cost tools with potential to support psychological well-being at the population level. However, the current evidence suggests that their impact remains largely confined to individual-level outcomes with limited examination of broader socio-emotional processes such as compassion and aggression. In addition, the generally high risk of bias observed across studies (e.g., reliance on self-report measures, limited use of active control groups, and high attrition rates) indicates that the certainty of the evidence remains limited.

Overall, this review indicates that the field is still in an early stage of development, characterized by strong emphasis on self-reported well-being and a relative neglect of interpersonal and process-oriented outcomes. Expanding the scope of research to include these dimensions is essential for exploring the full range of effects associated with mindfulness practice in digital contexts.

Furthermore, future research should prioritize more rigorous and ecologically valid methodological approaches. Specifically, randomized controlled trials with active control conditions, larger and more diverse samples (including non-Western and Spanish-speaking populations), and longer follow-up periods are needed. In addition, the incorporation of behavioral and ecological momentary assessment measures, alongside self-report instruments, would help clarify not only whether these interventions are effective, but also how and under which conditions they produce change. Finally, future work should examine mechanisms of change, such as emotional regulation, attentional control, and acceptance processes, to clarify not only whether these interventions are effective, but also how and under which conditions they produce change.

## Figures and Tables

**Table 1:** Selection codes.

(a) Measure: - Inclusion Criteria: Impact of MBIs (apps) on at least one variable of interest: well-being (subjective well-being, positive affect, negative affect, life satisfaction, psychological well-being, and flourishing), compassion or aggression (proactive and reactive). - Exclusion Criteria: Research does not include any of the three variables of interest. (b) Population: - Inclusion Criteria: Non-clinical population ≥ 18 years. - Exclusion Criteria: Clinical population (with a formal diagnosis) or <18 years. (c) Intervention: - Inclusion Criteria: Mindfulness as the basis of a secular intervention and not as an accessory or complement to a larger intervention package. Interventions must be applied via an app. - Exclusion Criteria: Interventions based on loving-kindness, compassion (CFT), self-compassion (MSC), cognitive therapy (MBCT), or other meditative practices. Apps used only as support for another modality (face-to-face, web, etc.). (d) Type of research: - Inclusion Criteria: Primary experimental empirical studies with at least 2 randomised comparison groups (active or passive control). Quantitative or mixed methodology. Indexed articles. - Exclusion Criteria: Empirical syntheses (systematic reviews, meta-analyses, scoping reviews, etc.) and protocols. Designs without 2 randomised groups or results. Purely qualitative studies. Grey literature.

*Note*. This table shows the criteria used for article selection.

**Table 2:** Review and Description of Included Studies (N = 23).

Study	Population (n)	App (n)	Control (n)	Session (min)	Duration	Outcome	Measuring of Instrument	Attrition ^1^ %	Effect Sizes (95% CIs if Applicable)
Bostock et al. (2019) [47]	Employees (238)	Headspace (128)	Waiting list (110)	45 (10–20 min)	8 w ^2^	W ^3^	WEMWBS ^4^	4%	Time-Group Interaction: *η*2: 0.03 **
Carissoli et al. (2021) [48]	Pregnant (108)	BenEssere Mamma (57)	PC ^5^ (51)	20 (3–20 min)	4 w	PsyW ^6^	PWBS ^7^	31%	Autonomy: *η*2: 0.032; Environmental mastery: *η*2: 0.00; Personal growth: *η*2: 0.00; Positive Relationships: *η*2: 0.030; Purpose in life: *η*2: 0.00; Self-acceptance: *η*2: 0.001. Time-Group Interaction: Autonomy: *η*2: 0.052; Environmental mastery: *η*2: 0.001; Personal growth: *η*2: 0.023; Positive relationships: *η*2: 0.013; Purpose in life: *η*2: 0.037; Self-acceptance: *η*2: 0.013
Champion et al. (2018) [49]	General population (74)	Headspace (38)	Waiting list (36)	30 (10 min–20 min)	30 d ^8^	LS ^9^	SWLS ^10^	16%	Inter-group: *d*: 0.60 ** (0.08–1.12)
Conley et al. (2024) [50]	Students (145)	Headspace (104)	Waiting list (41)	56 (30)	8 w	Aff ^11^; Hsu ^12^; C ^13^	PANAS ^14^; SHS ^15^; CLS ^16^	5%	Time-Group interaction: Aff+: *η*2: 0.08 ***; Aff-: *η*2: 0.03; SHS: *η*2: 0.05 **; CLS: *η*2: 0.02
Economides et al. (2018) [51]	General population (160)	Headspace (41)	Headspace-audible book (28)	10 (10 min)	––^17^	Aff	SPANE ^18^	57%	Inter-group: d: 0.47 ** (−1.92 a 2.87)
Fitzhugh et al. (2024) [52]	Police (1301)	Headspace (255); Mindfit-Cop (204)	Waiting list (350)	––	24 w	W; LS	WEMWBS; LS (UK ONS) ^19^	55%	W10w: Headspace: *B*: 2.62 * (0.04–4.87); Mindfit-Cop: *B*: 2.43 (−0.04–4.88). LS10w: Headspace: *B*: 1.47 ** (0.51–2.43); Mindfit-Cop: *B*: 1.22 * (0.16–2.28). W24w: Headspace: *B*: 4.65 *** (2.06–7.24); Mindfit-Cop: *B*: 4.00 ** (1.08–6.90). LS24w: Headspace: *B*: 1.57 ** (0.55–2.59); Mindfit-Cop: *B*: 1.57 ** (0.37–2.73).
Flett et al. (2018) [53]	Students (210)	Headspace (72); Smiling Mind (63)	Evernote (75)	10 (10 min)	10 d	Flo ^20^	FS ^21^	18%	App vs. Control: Headspace: *g de Hedges*: 0.08; Smiling Mind: *g de Hedges*: 0.12
Golec et al. (2024) [54]	General population (244)	–– (103)	Waiting list (116)	42 (30 min)	6 w	LS	SWLS	10%	Δ*R*2: 0.14
Howells et al. (2016) [45]	General population (194)	Headspace (57)	Catch Notes (64)	10 (10 min)	10 d	LS; Aff; Flo	SWLS; PANAS; FS	38%	SWLS: *η*2: 0.003; Aff +: *η*2: 0.071 **; Aff −: *η*2: *η2*:0.010; *FS*: *η*2: 0.006.
Keng et al. (2022) [55]	General population (80)	Headspace (40)	Lumosity (40)	10 (10 min)	10 d	SW ^22^	PWI ^23^	1%	*f*^2^: 0.03
Levin et al. (2020) [56]	Students (23)	Stop, Breathe & Think (10)	Waiting list (13)	24 (10 min)	4 w	W	MHCSF27 ^24^	30%	Pre-post App vs. Control: *g*: 0.52 (IC 90%, −0.31–1.41)
Lim et al. (2015) [41]	Students (56)	Headspace (27)	Lumosity (29)	14 (12 min)	3 w	C	NBM ^25^	23%	App vs. Control: *ϕ* = 0.27 *
Lindsay et al. (2018) [57]	General population (153)	Created for the study: MA ^26^ (58); MO ^27^ (58)	MyTime (37)	14 (20 min)	14 d	Aff	EMA ^28^ y DA ^29^	2%	DA: MA vs. MO: *g de Hedges*: 0.46 *; MA vs. Control: *g de Hedges*: 0.71 **; MO vs. Control: *g de Hedges*: 0.25. EMA: MA vs. MO: *g de Hedges*: 0.41 *; MA vs. Control: *g de Hedges*: 0.66 **; MO vs. Control: *g de Hedges*: 0.25.
Mak et al. (2018) [58]	General population (2161)	Living With Heart: MBP ^30^ (703); SCP ^31^(705)	CBP ^32^ (753)	28 (10–15 min)	4 w	SW	WHO-5 ^33^	76%	4 w: MBP: *d*: 0.31; SCP: *d*: 0.40; Control: *d*: 0.36.
Noone & Hogan, (2018) [59]	Students (91)	Headspace (43)	Sham meditation (48)	30 (10 min)	6 w	Aff; W	PANAS and WEMWBS	22%	––
O’Donnell et al. (2023) [60]	General population (100)	Insight Timer (51)	Waiting list (49)	30 (10 min)	30 d	SW	WHO-5	54%	Control vs. Intervention: *d*: 0.88 **
Schaller & Karing (2024) [61]	Students (89)	7Mind (30)	Nature (30)	7 (7 min)	7 w	LS	LSS-7 ^34^	19%	Time-Group interaction: *η*2: 0.0*12*
Schulte-Frankenfeld, & Trautwein, (2022) [62]	Students (99)	Balloon (50)	Waiting list (49)	57 (10 min)	8 w	LS	LSS ^35^	35%	LSS total: *η*2: 0.034; LSS actual: *η*2: 0.029; LSS past: *η*2: 0.016
Taylor et al. (2022) [63]	Health workers (2182)	Headspace (1095)	Moodzone (1087)	30 (10 min)	30 d	W	SWEMWBS ^36^	35%	30 d: *g de Hedges*: 0.07 *; 4 m: *g de Hedges*: 0.19 ***
Walsh et al. (2019) [64]	Students (108)	Wildflowers (58)	2048 (50)	21 (10 min)	3 w	PsyW	PWBS	20%	Acceptation: *r*: 0.15; Awareness: *r*: 0.14; Openness: *r*: 0.26 *. Time-Group interaction: Acceptation: *r*: 0.21; Awareness: *r*: 0.10; Openness: *r*: −0.05
Xu et al. (2022) [65]	Emergency workers (148)	Headspace (74)	Waiting list (74)	28 (10 min)	4 w	W	WEMWBS	30%	Within-group after intervention: Headspace: *d*: 0.56; Waiting list: *d*: 0.49. Time-Group interaction: *d*: 0.21. Time: *d*: 11.24 ***
Yang et al. (2018) [66]	Students (88)	Headspace (45)	Waiting list (43)	30 (10–20 min)	30 d	W	GWBS ^37^	8%	––
Yoon et al. (2022) [67]	Employees (45)	InMind (22)	Waiting list (23)	28 (10 min)	4 w	W	COMOSWB ^38^	7%	Time-Group interaction: *η*2: 0.09 *. Time: 4 s: *d*: 0.54 *

* *p* < 0.05; ** *p* < 0.01; *** *p* < 0.001; ^1^ Attrition rates as calculated by the current authors (based on the data reported in the studies); ^2^ Weeks; ^3^ Measure of general well-being (hedonic + eudaimonic); ^4^ WEMWBS: Warwick-Edinburgh Mental Wellbeing Scale; ^5^ Prenatal care; ^6^ Measure of psychological well-being; ^7^ PWBS: Psychological Well-Being Scale; ^8^ Days; ^9^ Measure of life satisfaction; ^10^ SWLS: Life Satisfaction Scale; ^11^ Measure of affection; ^12^ Measure of subjective happiness; ^13^ Measure of compassion; ^14^ PANAS: Positive and Negative Affect Scale; ^15^ SHS: Subjective Happiness Scale; ^16^ CLS: Compassionate Love Scale; ^17^ Data not reported; ^18^ SPANE: Scale of Positive and Negative Experiences; ^19^ LS (UK ONS): Life satisfaction. Office for National Statistics of the United Kingdom; ^20^ Measure of flourishing; ^21^ FS: Flourishing Scale; ^22^ Measure of subjective well-being; ^23^ PWI: Personal Well-Being Index; ^24^ MHCSF27: Mental Health Continuum Short Version; ^25^ NBM: Naturalistic Behavioral Measure; ^26^ MA: Mindfulness with instruction in both monitoring and acceptance; ^27^ MO: Mindfulness with instruction in monitoring only; ^28^ EMA: Ecological Momentary Assessment; ^29^ DA: Diary of Affect; ^30^ Mindfulness based program; ^31^ Self-compassion program; ^32^ Behavioral psychoeducation program; ^33^ WHO-5: World Health Organization Well-Being Index-5; ^34^ Life Satisfaction Scale-7 items Dalbert; ^35^ LSS: Questionnaire for Assessing Happiness (German: Lebensglückskal. Study with German speakers); ^36^ SWEMWBS: Warwick-Edinburgh Mental Wellbeing Scale. Abbreviated version; ^37^ GWBS: General Welfare Scale; ^38^ COMOSWB: Concise Measure of Subjective Well-being.

## Data Availability

No new data were created or analyzed in this study.

## Supplementary Materials

The following supporting information can be downloaded at: https://www.mdpi.com/article/10.3390/healthcare14111512/s1, Table S1: Checklist Prisma 2020. Table S2: Abstracts Checklist Prisma 2020. Figure S1: Risk of bias assessment.
